# Description of Streptomyces explomaris sp. nov., isolated from the coastal soil rhizosphere of Juniperus excelsa and reclassification of Streptomyces libani as a later heterotypic synonym of Streptomyces nigrescens

**DOI:** 10.1099/ijsem.0.006711

**Published:** 2025-05-30

**Authors:** Wei Shu, Christian Rückert-Reed, Olexandr Gromyko, Stepan Tistechok, Jörn Kalinowski, Andriy Luzhetskyy, Christoph Wittmann

**Affiliations:** 1 Institute of Systems Biotechnology, Saarland University, Saarbrücken, Germany; 2 Bielefeld University, CeBiTec, Technology Platform Genomics, Bielefeld, Germany; 3 Department of Genetics and Biotechnology, Ivan Franko National University of Lviv, Lviv, Ukraine; 4 Microbial Culture Collection of Antibiotic Producers, Ivan Franko National University of Lviv, Lviv, Ukraine; 5 Department of Pharmacy, Pharmaceutical Biotechnology, Saarland University, Saarbrücken, Germany

**Keywords:** actinomycete, biosynthetic gene cluster (BGC), marine coastal soil, *Streptomyces*, taxogenomic analysis, taxonomic analysis

## Abstract

The strain Je 1-4^T^ was isolated from the coastal rhizosphere soil of *Juniperus excelsa* M. Bieb. (Crimean Peninsula). Phylogenetic studies based on the 16S rRNA gene sequence revealed that Je 1-4^T^ is phylogenetically close to *Streptomyces libani* subsp. *libani* NBRC 13452^T^ (JCM 4322) and *Streptomyces nigrescens* NBRC 12894^T^ (DSM 40276) with sequence similarities of 99.86 to 99.93%. The genome of strain Je 1-4^T^ consisted of a linear chromosome with a size of 8.9 Mbp and a G+C content of 70.9mol%. Digital DNA–DNA hybridization (dDDH) analysis showed that Je 1-4^T^ is distinct from both *S. libani* subsp. *libani* and *S. nigrescens* (dDDH values of 66.6% and 66.7 %, respectively), while the latter two strains likely represent the same species (dDDH value of 92.0%). The predominant fatty acids in the strains were iso-C_16:0_, anteiso-C_17:0_ and anteiso-C_15:0_, and the major menaquinones were MK9 H6 and MK9 H8. Based on these genomic and phenotypic data, strain Je 1-4^T^ represents a novel species of *Streptomyces*, for which the name *Streptomyces explomaris* sp. nov. is proposed. The type strain is Je 1-4^T^ (=DSM 117375^T^=LMG 33490^T^). Additionally, we propose that *S. libani* subsp. *libani* Baldacci and Grein 1966 (Approved Lists 1980) is a later heterotypic synonym of *S. nigrescens* (Sveshnikova 1957) Pridham et al. 1958 (Approved Lists 1980).

## Data Summary

External data to this work comprising the deposited genome sequence, 16S rRNA gene sequence and raw read data of *Streptomyces explomaris* sp. nov. have been deposited in GenBank and the Sequence Read Archive under the accession numbers CP106840, OQ740289 and PRJNA883639, respectively. The sequences of the genome (CP114203) and the plasmid (CP114204) of *Streptomyces nigrescens* NBRC 12894^T^ have been deposited to GenBank. The supplementary data file contains Fig. S1, showing four phylogenetic trees inferred with mega 11 from multilocus sequence typing data using different approaches. The authors confirm that all other data have been provided within the article.

## Introduction

Actinomycetes, particularly *Streptomyces*, are significant producers of secondary metabolites, accounting for ~50% of all known microbial natural products [1]. Many of these metabolites possess remarkable properties, including antibacterial, anticancer and antiviral activities [2,6]. Consequently, the isolation, taxonomic description and further investigation of new *Streptomyces* species garner significant attention [7]. Various *Streptomyces* isolates have been obtained from diverse environments. Notably, marine ecosystems are receiving increasing attention as significant habitats for *Streptomyces* [8]. The unique characteristics of marine environments, such as high salinity, oligotrophy, high hydrostatic pressure, low pH and low temperature [9], suggest that marine *Streptomyces* isolates may exhibit distinct physiological and biochemical traits and produce metabolites with novel structures and biological activities [10,12]. Recent examples of *Streptomyces* isolated from marine ecosystems include *Streptomyces bathyalis* [13], *Streptomyces lydicamycinicus* [14], *Streptomyces fradiae* MM456M-mF7 [15], *Streptomyces oceani* [16] and *Streptomyces bohaiensis* [17].

In the present study, strain Je 1-4^T^ was isolated from the coastal rhizosphere soil of *Juniperus excelsa* M. Bieb. on the Crimean Peninsula. 16S rRNA gene sequence similarity studies revealed that Je 1-4^T^ is phylogenetically close to *Streptomyces libani* subsp. *libani* NBRC 13452^T^ (JCM 4322) and *Streptomyces nigrescens* NBRC 12894^T^ (DSM 40276). Based on a detailed investigation of its taxonomic status, strain Je 1-4^T^ represents a novel species of *Streptomyces*, for which the name *Streptomyces explomaris* sp. nov. is proposed.

## Methods

### Genome sequencing

Genomic DNA of strain Je 1-4^T^ was isolated using the NucleoSpin Microbial DNA kit (Macherey and Nagel, Düren, Germany). Subsequently, DNA reads were generated via Nanopore and Illumina sequencing. For library preparation, the TruSeq DNA PCR-Free High-Throughput Library Prep Kit (Illumina, San Diego, CA, USA) and the SQK-LSK109 Sequencing Kit (Oxford Nanopore Technologies, Oxford, UK) were utilized without prior DNA shearing. Short reads were generated using a 2×300-nucleotide run (MiSeq Reagent Kit v3, 600 cycles, Illumina), while long reads were produced on a GridION platform using an R9.4.1 flow cell (Nanopore). Base calling and demultiplexing were performed with GUPPY v5.0.16 [18] using the super-accurate basecalling model. Assemblies were conducted using Flye v.2.9 for Nanopore data [19] and Newbler v2.8 for Illumina data [20]. The Flye-based assembly was polished using Medaka v1.5.0 [21] and Pilon v1.22 [22], with Bowtie2 [23] employed for mapping. The Flye and Newbler assemblies were then combined using Consed v28.0 [24]. The resulting single contig, representing the linear genome, was checked with busco [25] and CheckM [26] for completeness and contamination, showing that the genome is indeed complete (busco: 98.7% complete, 0.8% fragmented, 0.4% missing; CheckM: 99.1% completeness) and free of contaminations (CheckM: 0.71% contamination). The final genome was annotated with the PGAP pipeline [27,28] and submitted to GenBank (CP106840).

### 16S rRNA gene classification and sequence verification

The sequences of the seven 16S rRNA genes (SLV14_001993, SLV14_002131, SLV14_003358, SLV14_004352, SLV14_004947, SLV14_006120 and SLV14_006859) were extracted from the complete genome sequence and individually compared against the EzBioCloud 16S database [29]. From those seven sequences, the 16S rRNA gene consensus sequence was derived, resulting in two wobble bases at positions 179 ([CT]=Y) and 425 ([AG]=R). The consensus sequence was deposited in GenBank under the accession number OQ740289.

The 16S rRNA gene sequence was verified by performing a PCR reaction with 30 cycles using Q5 High-Fidelity DNA Polymerase (New England Biolabs) with the 27F (AGRGTTYGATYMTGGCTCAG) and 1492R (RGYTACCTTGTTACGACTT) primers [30] using mycelial material as a template and extending the initial denaturation step to 15 min. The obtained product was purified using Ampure XP beads, sequenced on an ABI 3730xl DNA Analyser (Applied Biosystems) and compared to consensus sequence using SnapGene v5.0.8 (Dotmatics).

### Genome-based phylogeny

Average Nucleotide Identity (ANI) values were calculated using OrthoANIu [31]. The genome sequence data were uploaded to the Type (Strain) Genome Server (TYGS) (https://tygs.dsmz.de) for whole-genome-based taxonomic analysis, which includes recent methodological updates and features [32]. TYGS provided information on nomenclature, synonymy and associated taxonomic literature via its sister database, the List of Prokaryotic names with Standing in Nomenclature (https://lpsn.dsmz.de) [32]. Results from the TYGS analysis were provided on 22 July 2024.

The TYGS analysis involved several steps. The closest type-strain genomes were determined through two complementary approaches. Firstly, all user genomes were compared against all type strain genomes in the TYGS database using the MASH algorithm, identifying the ten type strains with the smallest MASH distances per user genome [33]. Secondly, an additional set of ten closely related type strains was determined through 16S rRNA gene sequences, which were extracted from the user genomes using RNAmmer [34] and subsequently BLASTed [35] against the 16S rRNA gene sequences of 21 376 type strains in the TYGS database. This analysis identified the best 50 matching type strains based on bit score, followed by precise distance calculations using the Genome blast Distance Phylogeny (GBDP) approach under the ‘coverage’ algorithm and distance formula d5 [36]. The obtained distances identified the ten closest type strain genomes for each user genome.

For phylogenomic inference, all pairwise comparisons among the set of genomes were conducted using GBDP, with accurate intergenomic distances inferred under the ‘trimming’ algorithm and distance formula d5, calculating 100 distance replicates each [36]. The digital DDH (dDDH) values and confidence intervals were calculated using the Genome-to-Genome Distance Calculator with recommended settings (GGDC 4.0) [36]. The resulting intergenomic distances were used to infer a balanced minimum evolution tree with branch support via FastME 2.1.6.1, including Subtree Pruning and Regrafting (SPR) post-processing [37]. Branch support was inferred from 100 pseudo-bootstrap replicates each. The trees were rooted at the midpoint [38] and visualized with PhyD3 [39].

Type-based species clustering using a 70% dDDH radius around each type strain was conducted as described previously [32], with subspecies clustering done using a 79% dDDH threshold [40]. For multilocus sequence typing (MLST)-based phylogenetic analyses, mega 11 was employed [41]. This software facilitated alignment using clustal, focusing on the *atpD*, *gyrB*, *recA*, *rpoB* and *trpB* genes listed for *Streptomyces* spp. in PubMLST [42]. Additionally, mega 11 was used to construct the corresponding phylogenetic trees. The methods employed for this analysis included minimum evolution (ME), maximum likelihood (ML), maximum parsimony (MP) and neighbour-joining (NJ). The mega Analysis Option parameters used for clustal alignment and tree generation are listed in Table S2, Table S3, Table S4, Table S5 and Table S6 (available in the online Supplementary Material).

### *In silico* prediction of secondary metabolite clusters

Secondary metabolite biosynthesis clusters were predicted using the antiSMASH web service [43], version 8.dev-0de8f3d7, with the annotated GenBank file as input.

### Morphological analysis

Morphological observations of substrate and aerial mycelium were conducted after a 14-day incubation at 30 °C on International *Streptomyces* Project (ISP) solid media 1–7 [44]. The colours of the mycelium and diffusible pigments were assessed using RAL-code colour chips [45]. Spore chain morphology and spore surface ornamentation were examined using scanning electron microscopy on sporulating cultures grown on an ISP 3 medium for 28 days at 30 °C. Sample preparation was adapted from previous work [46]. For this purpose, a section (0.5 cm^2^) of agar with bacterial lawn was cut off, fixed with 5% glutaraldehyde for 2 h at 4 °C, twice resuspended in 0.1 M phosphate buffer (pH 7.2), thoroughly mixed for 10 min and collected by centrifugation (5 min, 5000 ***g***, RT). The sample was dehydrated using a series of ethanol solutions with increasing concentration (35%, 50%, 75%, 95% and 100%), washed three times with pure ethanol, freeze-dried, sputtered with gold and subsequently examined (ZEISS Sigma VP FEG-SEM).

### Physiological and biochemical studies

Growth was assessed at 25, 28, 30, 37 and 45 °C. For this purpose, strains were incubated in shake flasks containing GYM medium on an orbital shaker at 230 r.p.m. for 7 days. The GYM medium contained per litre: 4 g glucose, 4 g yeast extract (BD Difco, Becton Dickinson, Heidelberg, Germany) and 10 g malt extract (BD Difco, Becton Dickinson). To study the effects of pH and salt on growth, the GYM medium was adjusted to different pH values (3.0, 4.0, 5.0, 6.0, 7.0, 8.0, 9.0 and 10.0) and various NaCl concentrations (0, 2.5, 5.0, 7.5 and 10.0%). Strains were grown for 7 days in a miniaturized incubator (Biolector, Beckmann Coulter, Baesweiler, Germany) using 48-well microtiter plates, with growth monitored online [47]. Carbohydrate assimilation was studied at 30 °C on ISP 9 agar supplemented with 1% of glucose, xylose, fructose, cellulose, mannitol, inositol, sucrose, arabinose, rhamnose or raffinose [44]. Biochemical characteristics were evaluated using commercial test kits (API ZYM, API 50CH, bioMérieux, Nürnberg, Germany). Antibiotic susceptibility was investigated using disc-diffusion plate tests, with strains incubated for 7 days at 30 °C on ISP 2 agar [48]. The following antibiotics were evaluated: ampicillin (10 µg per disc), erythromycin (15 µg per disc), gentamicin (30 µg per disc), tetracycline (30 µg per disc), vancomycin (30 µg per disc), cefotaxime (30 µg per disc), rifampicin (5 µg per disc) and penicillin G (6 µg per disc) [13].

### Chemotaxonomical analysis

Biomass was harvested from liquid cultures after incubation in GYM medium for 48 h on an orbital shaker at 230 r.p.m. and 28 °C. The analysis of cellular fatty acids, respiratory quinones, polar lipids, mycolic acids, whole-cell sugars and diaminopimelic acid isomers was performed by the DSMZ Identification Service (Leibniz Institute DSMZ, Braunschweig, Germany).

## Results and discussion

### Genome sequencing and genome-based phylogeny

The genome assembly of strain Je 1-4 ^T^ consisted of a single contig, representing the linear genome. Its seven 16S rRNA gene sequences were individually compared against the EzBioCloud 16S database [29], revealing the highest similarities with *S. nigrescens* NBRC 12894^T^ (99.86 –99.93 %), *S. libani* subsp. *libani* NBRC 13452^T^ (99.86 –99.93 %), *Streptomyces angustmyceticus* NRRL B 2347^T^ (99.79 %–99.93 %), *Streptomyces tubercidicus* DSM 40261^T^ (99.79 –99.86 %) and *S. lydicamycinicus* NBRC 110027^T^ (99.72 –99.86 %). Given that 16S rRNA gene similarity alone is insufficient for proper classification, a genome-based phylogeny analysis was performed using the TYGS [32]. The sequences of the genome and the plasmid of *S. nigrescens* NBRC 12894^T^, not available on the server, were determined using the previously described workflow and submitted to GenBank (CP114203 and CP114204).

**Fig. 1.**
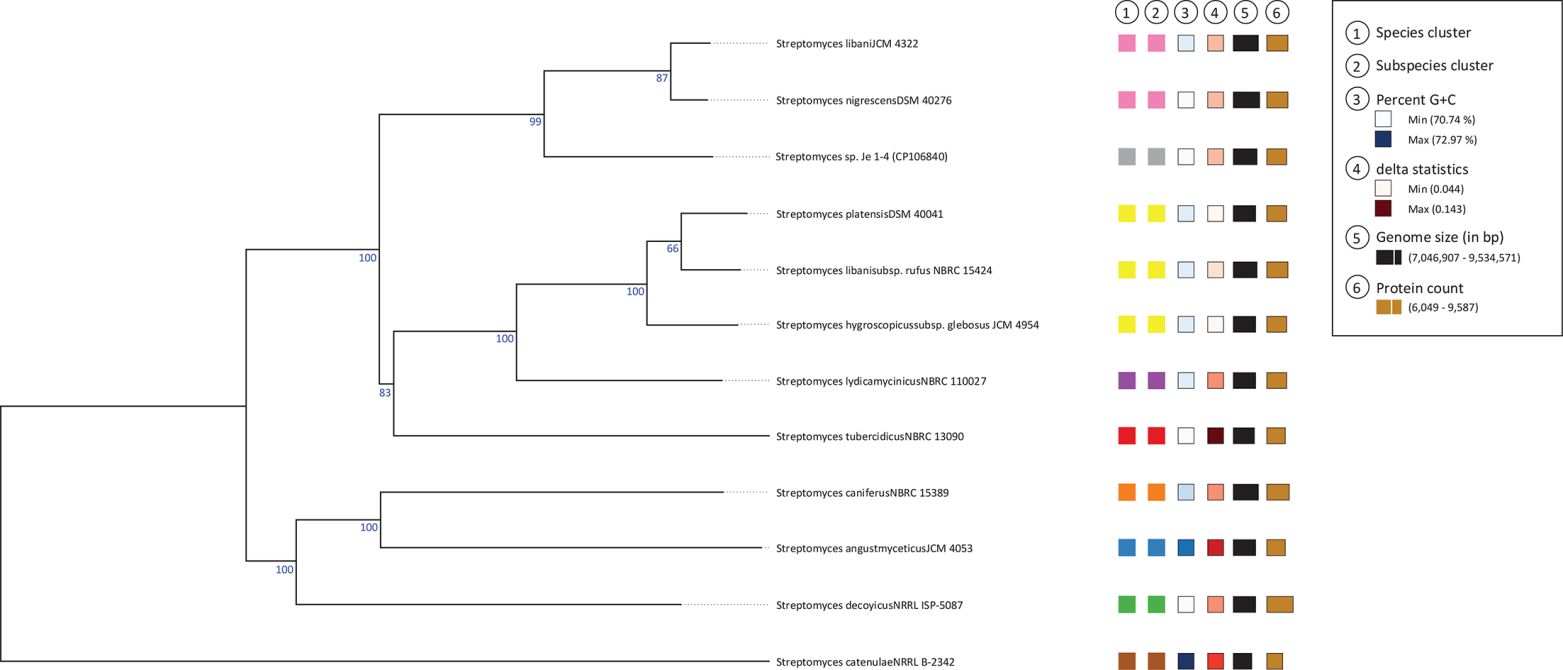
Phylogenetic tree inferred with FastME 2.1.6.1 from GBDP distances calculated from genome sequences. Branch lengths are scaled in terms of the GBDP distance formula d5. The numbers above branches are GBDP pseudo-bootstrap support values >60% from 100 replications, with an average branch support of 87.5%. The tree was rooted at the midpoint.

dDDH of the genome data indicated that while strain Je 1-4 ^T^ is related to *S. libani* subsp. *libani* and *S. nigrescens*, it is distinct from both, with dDDH values of 66.6% and 66.7 %, respectively, which are below the 70% cutoff used for species delineation (Table 1, Fig. 1). Interestingly, the analysis suggested that *S. libani* subsp. *libani* JCM 4322^T^ and *S. nigrescens* DSM 40276^T^ likely represent the same species, with a dDDH value of 92.0%.

**Table 1.** Pairwise comparison of the genome of strain Je 1-4^T^ against similar type-strain genomes The dDDH values are provided along with their confidence intervals (C.I.).

Strains	dDDH (%)	C.I.	ANI (%)
*Streptomyces libani* JCM 4322	66.6	75.6–83.0	95.9
*S. nigrescens* DSM 40276	66.7	71.6–79.2	95.9
*Streptomyces platensis* DSM 40041	45.8	59.7–67.1	91.8
*Streptomyces libani* subsp. *rufus* NBRC 15424	45.9	59.0–66.3	91.9
*Streptomyces hygroscopicus* subsp. *glebosus* JCM 4954	45.7	59.1–66.5	91.7
*S. lydicamycinicus* NBRC 110027	46.8	63.2–70.7	92.1
*S. tubercidicus* NBRC 13090	44.2	52.3–59.3	91.3
*Streptomyces caniferus* NBRC 15389	37.4	44.9–51.8	88.8
*S. angustmyceticus* JCM 4053	36.0	46.0–52.9	88.4
*Streptomyces decoyicus* NRRL ISP-5087	39.4	43.4–50.2	89.6
*Streptomyces catenulae* NRRL B-2342	25.6	23.2–30.2	82.0

In addition to the dDDH distance-based ME tree, we calculated four phylogenetic trees based on a clustal alignment of the *atpD*, *gyrB*, *recA, rpoB* and *trpB* genes. The algorithms employed were ME, ML, MP, and NJ. Despite using only a fraction of the data (~2580 bp) compared to the dDDH calculation, all trees showed almost identical relationships (Figs 1 and S1). The only major discrepancy was observed with *Streptomyces decoyicus* NRRL ISP-5087^T^, which clustered with *Streptomyces caniferus* NBRC 15389^T^ and *S. angustmyceticus* JCM 4053^T^ in the dDDH-based tree, wherein it grouped with *Streptomyces* sp. Je 1-4^T^, *S. libani* JCM 4322^T^ and *S. nigrescens* DSM 40276^T^ in the MLST-based trees.

The genomic features of strain Je 1-4^T^ are consistent with those of many other *Streptomyces* spp. The linear chromosome is 8.9 Mbp in size with a G+C content of 70.9%. The strain contains no plasmids. The inverted terminal repeats at the ends of the chromosome are relatively small, measuring 36.4 kbp each. The annotation pipeline predicted a total of 7707 genes, with 7377 encoding functional proteins and 239 predicted to be pseudogenes. Additionally, 91 RNA genes were identified, comprising 67 tRNAs, 21 rRNAs organized in 7 operons and 3 ncRNAs.

The genome sequence was analysed using antiSMASH (version 8.dev-0de8f3d7) [43], revealing 35 putative biosynthetic gene clusters (BGCs) associated with the production of various secondary metabolites (Table S1). These BGCs include eight terpenes, three non-ribosomal peptides, three polyketides, two non-ribosomal peptide-polyketide hybrids and eight ribosomally synthesized and post-translationally modified peptides. Among the identified BGCs, eight demonstrated 100% similarity with known gene clusters responsible for the biosynthesis of antipain, lydicamycin, pseudouridinomycin, ulleungdin, spore pigment, the siderophore desferrioxamine B, the morphogen SapB and the compatible solute ectoine. Additionally, two BGCs exhibited over 50% similarity to gene clusters involved in hopene and caniferolide biosynthesis. Notably, more than half of the identified BGCs remain uncharacterized, highlighting the substantial biosynthetic potential of *S. explomaris*.

### Growth properties and utilization of carbon sources

All three strains grew well on ISP 2, ISP 3, ISP 4, ISP 5 and ISP 7 media. On ISP 6 medium, Je 1-4^T^ exhibited poor growth, while the other two strains did not grow at all. Je 1-4^T^ formed substrate mycelium on all tested media and aerial mycelium on all media except ISP 6. Generally, no diffusible pigments were detected. A summary of all observed morphological features is provided in Table 2.

**Table 2.** Phenotypic strain characteristics Strains 1: Je 1-4^T^; 2: *S. libani subsp. libani* NBRC 13452^T^; 3: *S. nigrescens* NBRC 12894^T^. All strains are positive for glucose, mannitol, fructose, xylose, sucrose, inositol and raffinose and negative for cellulose, arabinose and rhamnose. The capability to utilize other carbon sources differs between the strains, indicated as + (positive) and – (negative). Abbreviations: DPG: diphosphatidylglycerol; PE: phosphatidylethanolamine; PG: phosphatidylglycerol; PI: phosphatidylinositol; APL: aminophospholipid; GL: glycolipid; PL: phospholipid.

Characteristic	Strain 1	Strain 2	Strain 3
**Cultivation**			
ISP 2 agar			
Growth	Good	Good	Good
Colour of colony	Cement grey	Cement grey	Silver grey
ISP 3 agar			
Growth	Good	Good	Good
Colour of colony	Moss grey	Silver grey	Silver grey
ISP 4 agar			
Growth	Good	Good	Good
Colour of colony	Black grey	Green grey	Green grey
ISP 5 agar			
Growth	Good	Good	Good
Colour of colony	Cement grey	Telegrey	Traffic grey
ISP 6 agar			
Growth	Sparse	None	None
Colour of colony	Beige		
ISP 7 agar			
Growth	Good	Good	Good
Colour of colony	Beige	Traffic white	Green grey
**Carbon source utilization**			
*N*-Acetylglucosamine	+	+	−
d-Cellobiose	+	−	−
l-Fucose	+	+	−
Gentiobiose	+	+	−
Optimum growth (℃)	25–30	25–37	25–37
Optimum growth (pH)	6.0–8.0	6.0–8.0	6.0–8.0
Optimum growth (% NaCl)	0–2.5	0–5.0	0–7.5
**Menaquinones**			
MK9 H_4_	+	+	+
MK9 H_6_	+	+	+
MK9 H_8_	+	+	+
**Fatty acid content (%**)			
* iso*-C_14:0_	2.3	3.3	4.3
* iso*-C_15:0_	7.0	7.4	6.5
* anteiso*-C_15:0_	14.3	13.2	11.6
C_15:0_	1.2	−	−
* anteiso*-C_15:0_ 2OH	1.4	1.1	−
* so*-C_16:1_ h	2.3	3.1	3.3
* iso*-C_16:0_	24.1	28.3	35.4
C_16:1_ ω7 c	4.0	3.8	3.4
C_16:0_	5.8	5.2	6.0
* iso*-C_17:1_ ω7 c	4.5	4.2	3.1
* anteiso*-C_17:1_ ω7 c	4.2	3.6	2.8
* iso*-C_17:0_	5.7	5.3	4.4
* anteiso*-C_17:0_	15.6	12.5	10.3
C_17:0_ cyclo ω7 c	1.3	1.3	1.3
* so*-C_18:1_ h	1.0	1.1	1.1
**Polar lipids**	DPG, PE, PG, PI, 2APLs, 2PLs, GL	DPG, PE, PG, PI, 2APLs, PL, GL	DPG, PE, PG, PI, 3APLs, PL, GL

**Fig. 2.**
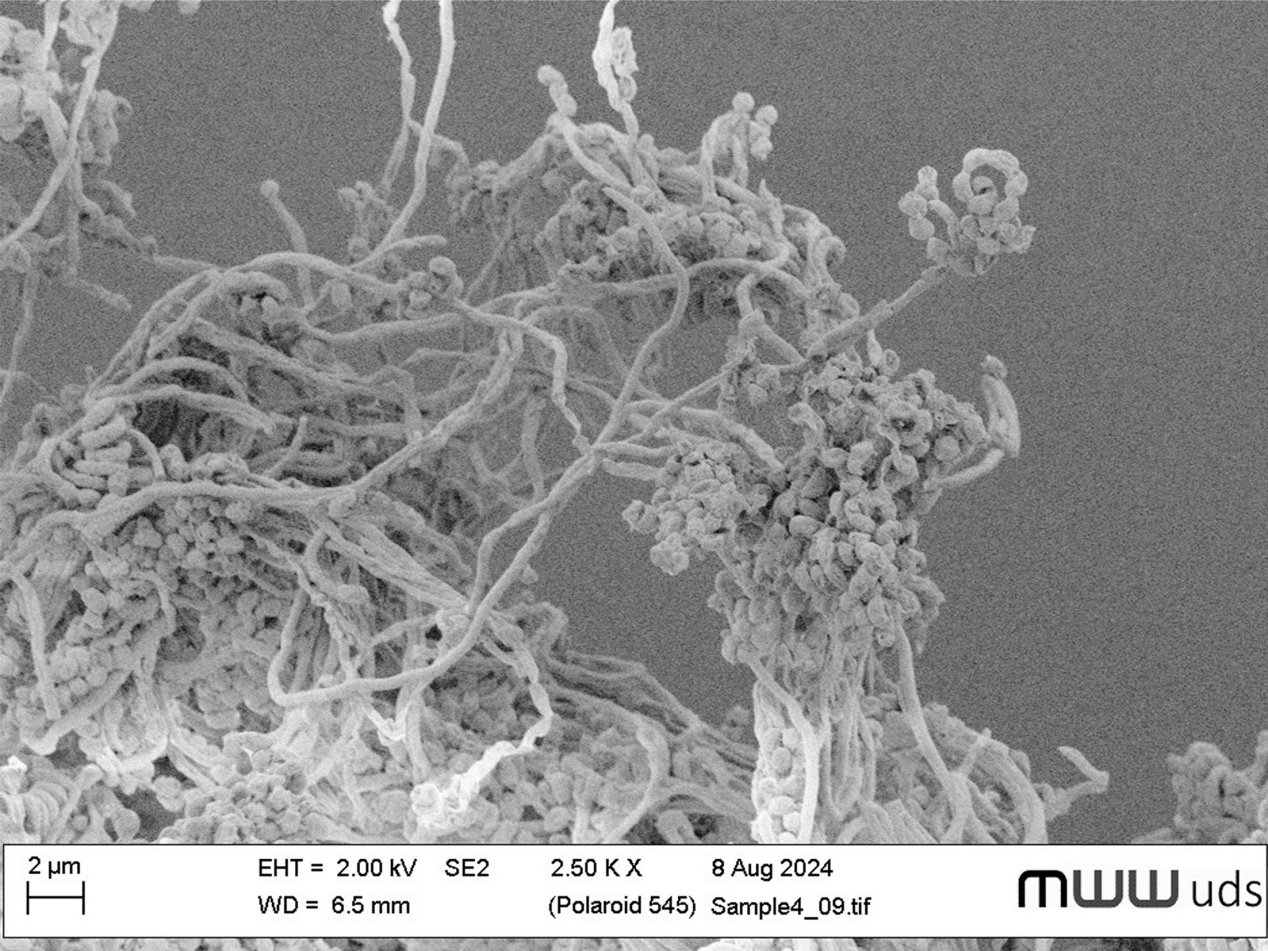
Scanning electron micrograph of strain Je 1-4^T^. Prior to analysis, the strain was incubated on ISP 3 agar for T days at 30 ℃.

Je 1-4^T^ formed straight to flexuous, partially spiral spore chains on solid media, with smooth surfaces (Fig. 2). The spores were oval to cylindrical in shape, arranged in long chains and each contained more about 10 to more than 25 spores [49]. The mature spores were ~0.5–1.0 mm in diameter, and the length was between 0.8 and 1.0 mm.

Je 1-4^T^ grew at temperatures ranging from 25 to 45 °C, with optimal growth observed at 25–30 °C. In comparison, *S. libani* subsp. *libani* and *S. nigrescens* showed optimal growth between 25 and 37 °C. Je 1-4^T^ grew at pH levels between 6 and 8, with an optimum pH of 7, and tolerated up to 2.5 % NaCl. The metabolic properties of Je 1-4^T^, in comparison to *S. libani* and *S. nigrescens*, are summarized in Table 2. According to the API 50 CH test, strain Je 1-4^T^ metabolized glycerol, d-arabinose, d-ribose, d-adonitol, d-galactose, d-glucose, d-fructose, d-mannose, inositol, d-mannitol, d-sorbitol, *N*-acetylglucosamine, d-cellobiose, d-maltose, d-lactose, d-melibiose, d-sucrose, d-trehalose, d-raffinose, starch, glycogen, xylitol, gentiobiose, d-turanose, l-fucose and d-arabitol. Enzymatic activities tested by the API ZYM kit indicated similar profiles for all three strains. Strain Je 1-4^T^ was susceptible to erythromycin, gentamicin, tetracycline, vancomycin and rifampicin, but not sensitive to ampicillin, cefotaxime and penicillin G, similar to its close phylogenetic neighbours.

### Chemotaxonomic characteristics

Chemotaxonomically, strain Je 1-4^T^ exhibited typical characteristics of the genus *Streptomyces*, including the presence of ll-diaminopimelic acid in the cell wall [50] and a predominant menaquinone composition [51]. Its whole-cell hydrolysate contained galactose and ribose as major whole-cell sugars, with minor amounts of glucose and mannose. Mycolic acids were not detected. The main menaquinones were MK9 H_6_ (66.6 %), MK9 H_8_ (16.6 %) and MK9 H_4_ (11.9 %). The fatty acid profile was characterized by *iso*-C_16:0_ (24.1 %), *anteiso*-C_17:0_ (15.6 %), *anteiso*-C_15:0_ (14.3 %), *iso*-C_15:0_ (7 %), C_16:0_ (5.8 %) and *iso*-C_17:0_ (5.7 %) as the predominant fatty acids. In comparison, *S. libani* and *S. nigrescens* presented a similar spectrum of menaquinones and fatty acids but with varying content. The identified polar lipids comprised diphosphatidylglycerol, phosphatidylethanolamine, phosphatidylglycerol, aminophospholipids, phosphatidylinositol and phospholipids (Table 2, File S1**,** Fig. S2).

### Reclassification of *S. libani*

Based on the high dDDH value of 92.0%, it can be argued that *S. libani* subsp. *libani* and *S. nigrescens* belong to the same genomospecies. A similar observation was previously made for the more distantly related *Streptomyces platensis*, *Streptomyces libani* subsp. *rufus* and *Streptomyces hygroscopicus* subsp. *glebosus*, the latter two of which were found to be heterotypic synonyms of the former [52]. Although our chemotaxonomic analyses revealed some minor differences between these two type strains, these are likely due to minor genetic variations and do not justify retaining them as separate species.

In such cases, Rule 24 of the Bacteriological Code applies, wherein the priority of names is determined by the date of original publication. In such a case, Rule 24 of the Bacteriological Code applies, in which the priority of names is determined by the date of the original publication. Therefore, we propose that *S. libani* subsp. *libani* Baldacci and Grein 1966 (Approved Lists 1980) is a later heterotypic synonym of *S. nigrescens* (Sveshnikova 1957) Pridham et al. 1958 (Approved Lists 1980).

### Description of *Streptomyces explomaris* sp. nov.

*Streptomyces explomaris* (ex.plo.ma’ris. N.L. gen. n. *explomaris*, of EXPLOMARE, named after the publicly funded project which enabled its taxonomic classification). This Gram-positive, aerobic actinomycete forms an extensively branched substrate mycelium that supports aerial hyphae differentiating into straight to flexuous spore chains. It grows within a temperature range of 25–45 °C, with optimal growth at 25–30 °C. It thrives in pH ranges from 6 to 8, optimally at pH 7, and can tolerate up to 2.5 % w/v sodium chloride. It produces several enzymes, including alkaline phosphatase, esterase (C4), esterase lipase (C8), leucine arylamidase, valine arylamidase, cystine arylamidase, acid phosphatase and naphthol-AS-BI-phosphohydrolase. It metabolizes glucose, mannitol, fructose, xylose, sucrose, inositol and raffinose but cannot utilize cellulose, arabinose and rhamnose. Chemotaxonomically, the cell wall peptidoglycan contains ll-diaminopimelic acid. Whole-cell hydrolysates primarily contain galactose and ribose. The major fatty acids are *iso*-C_16:0_, *anteiso*-C_17:0_ and *anteiso*-C_15:0_, while the predominant menaquinones are MK9 H_6_, MK9 H_8_ and MK9 H_4_. The polar lipid profile of *S. explomaris* includes diphosphatidylglycerol, phosphatidylethanolamine, phosphatidylglycerol, aminophospholipid, phospholipid and phosphatidylinositol. The genome of the type strain consists of a linear chromosome with a size of 8 866 687 bp and a G+C content of 70.9 mol%.

The type strain of *S. explomaris* is strain Je 1-4^T^, which was isolated from the coastal rhizosphere soil of *J. excelsa* M. Bieb. (Crimean Peninsula). This strain is stored in the Microbial Culture Collection of Antibiotic Producers of the Ivan Franko National University of Lviv under the collection number Lv 166. Additionally, it is deposited under the number DSM 117375^T^ at the Leibniz-Institute DSMZ - Deutsche Sammlung von Mikroorganismen und Zellkulturen GmbH (Braunschweig, Germany) and under the number LMG 33490^T^ at the Belgian Coordinated Collections of Microorganisms (BCCM/LMG, Gent, Belgium). The genome sequence, the 16S rRNA gene consensus sequence and the raw sequencing data are available via GenBank accessions CP106840 and OQ740289 as well as BioProject ID PRJNA883639.

## Supplementary material

10.1099/ijsem.0.006711 Uncited Supplementary Material 1.

## Abbreviations

ANI: average nucleotide identity
BGCs: biosynthetic gene clusters
dDDH: digital DNA–DNA hybridization
GBDP: Genome blast Distance Phylogeny
ISP: International *Streptomyces* Project
ME: minimum evolution
ML: maximum likelihood
MLST: multilocus sequence typing
MP: maximum parsimony
NJ: neighbour-joining
SPR: subtree pruning and regrafting
TYGS: Type (Strain) Genome Server
