# Supplementary material for: Description of Streptomyces explomaris sp. nov., isolated from the coastal soil rhizosphere of Juniperus excelsa and reclassification of Streptomyces libani as a later heterotypic synonym of Streptomyces nigrescens
Source: Int J Syst Evol Microbiol. 2025 May 30;75(5):006711. doi: 10.1099/ijsem.0.006711 (PMC12163732; doi:10.1099/ijsem.0.006711)
Supplement: Uncited Supplementary Material 1. [file ijsem-75-06711-s001.pdf]

Supplementary Material to

Description of *Streptomyces explomaris* sp. nov., isolated from the coastal soil rhizosphere of *Juniperus excelsa* and reclassification of *Streptomyces libani* as later heterotypic synonym of *Streptomyces nigrescens*

Wei Shu[1], Christian Rückert-Reed[2], Oleksandr Gromyko[3,4], Stepan Tistechok[3], Jörn Kalinowski[2], Andriy Luzhetskyy[5], and Christoph Wittmann[1*]

[1] Institute of Systems Biotechnology, Saarland University, Saarbrücken, Germany

[2] Bielefeld University, CeBiTec, Technology Platform Genomics, Bielefeld, Germany

[3] Department of Genetics and Biotechnology, Ivan Franko National University of Lviv, Lviv, Ukraine

[4] Microbial Culture Collection of Antibiotic Producers, Ivan Franko National University of Lviv, Lviv, Ukraine

[5] Department of Pharmacy, Pharmaceutical Biotechnology, Saarland University, Saarbrücken, Germany

*Corresponding author : Christoph Wittmann, christoph.wittmann@uni-saarland.de

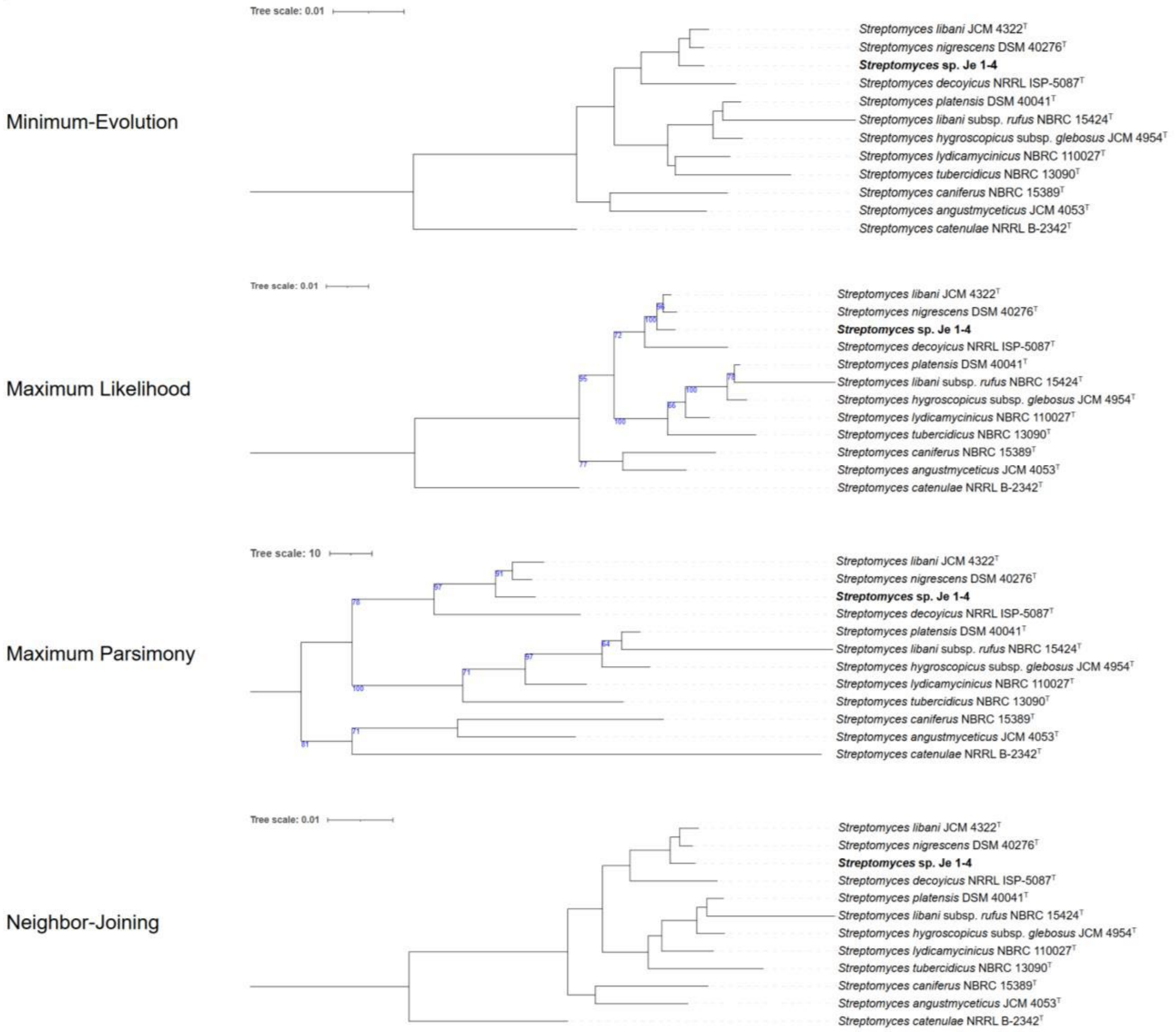

Figure S1. Calculation of four phylogenetic trees based on a CLUSTAL alignment of the *atpD, gyrB, recA*, *rpoB*, and *trpB* genes. The algorithms employed were Minimum-Evolution (ME), Maximum Likelihood (ML), Maximum Parsimony (MP), and Neighbor-Joining (NJ).

DPG = Diphosphatidylglycerol

PE = Phosphatidylethanolamine

PG = Phosphatidylglycerol

PI = Phosphatidylinositol

AGL = Aminoglycolipid

APL = Aminophospholipid

AL = Aminolipid

GL = Glycolipid

PL = Phospholipid

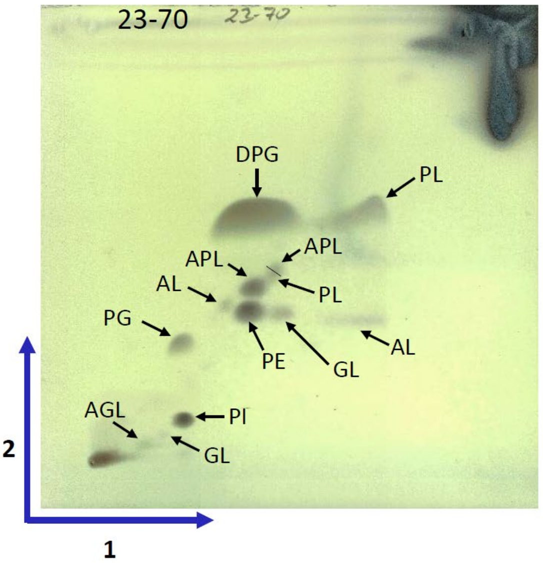

Figure S2. Polar lipid analysis of strain Je 1-4^{T} (DSMZ Identification Services)

Table S1. Biosynthetic gene clusters (BGCs) encoded in the genome strain Je 1-4^{T} as revealed by **ANTISMASH** analysis.

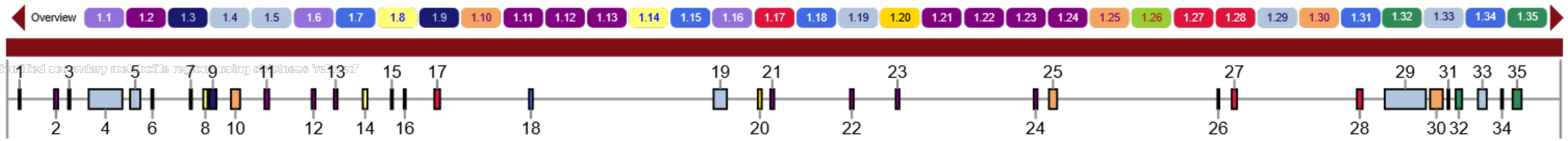

Region	Type	From	To	Confidence	Most similar known cluster	
Region 1	butyrolactone	64,149	75,069			
Region 2	terpene	266,230	289,505	Low	altemicidin/SB-203207/SB-203208	Alkaloid
Region 3	hydrogen-cyanide	346,625	359,565	Low	aborycin	RiPP
Region 4	terpene, T1PKS, PKS-like, NRPS-like, oligosaccharide	464,052	656,563	Medium	caniferolide A/caniferolide B/caniferolide C/caniferolide D	Polyketide:Modular type I polyketide
Region 5	other, NRPS, NRPS-like	700,466	758,707	High	antipain	NRP
Region 6	butyrolactone	822,187	833,170			
Region 7	RiPP-like	1,041,330	1,053,264			
Region 8	lanthipeptide-class-i	1,118,194	1,142,743			
Region 9	other, nucleoside	1,152,263	1,193,519	High	pseudouridimycin	Other:Nucleoside
Region 10	T1PKS, hglE-KS	1,276,250	1,328,406			
Region 11	terpene	1,467,685	1,494,372	Medium	hopene	Terpene
Region 12	terpene-precursor	1,737,340	1,758,392			
Region 13	terpene-precursor	1,863,613	1,884,665			
Region 14	lanthipeptide-class-i	2,028,763	2,055,238			
Region 15	RiPP-like	2,189,058	2,200,480			
Region 16	butyrolactone	2,262,874	2,273,851			
Region 17	NI-siderophore	2,437,320	2,470,014			
Region 18	triceptide	2,977,981	2,999,534			
Region 19	NRPS-like, NRPS, lassopeptide	4,031,623	4,107,356	High	ulleungdin	RiPP:Lassopeptide
Region 20	azole-containing-RiPP	4,284,288	4,306,556			
Region 21	terpene	4,355,427	4,377,661			
Region 22	terpene	4,810,555	4,831,328			
Region 23	terpene-precursor	5,071,381	5,092,394			
Region 24	terpene	5,859,494	5,880,555			
Region 25	T1PKS	5,946,350	5,992,691	Low	melanin	Other
Region 26	ectoine	6,908,408	6,918,824	High	ectoine	Other:Ectoine
Region 27	NI-siderophore	6,989,954	7,019,771	High	legonoxamine A/desferrioxamine B/legonoxamine B	Other
Region 28	NI-siderophore	7,704,179	7,737,036	Low	peucechelin	NRP
Region 29	T1PKS, NRPS, NRP-metallophore, melanin, aminopolycarboxylic-acid, hglE-KS, NRPS-like	7,863,355	8,098,138	High	lydicamycin	NRP+Polyketide:Modular type I polyketide
Region 30	T2PKS	8,122,151	8,194,666	High	spore pigment	Polyketide
Region 31	RiPP-like	8,221,582	8,231,809			
Region 32	NAPAA	8,268,355	8,305,026			
Region 33	lanthipeptide-class-iii, T3PKS	8,395,466	8,445,523	High	SapB	RiPP:Lanthipeptide
Region 34	RiPP-like	8,527,104	8,538,276			
Region 35	NRPS	8,593,273	8,644,594			

Table S2: clustal_align_nucleotide.mao

[MEGAinfo]
ver = 10200331-x86_64 MS Windows
[DataSettings]
datatype = snNucleotide
containsCodingNuc = False
missingBaseSymbol = ?
identicalBaseSymbol = .
gapSymbol = -
[ProcessTypes]
ppAlign = true
ppClustalW = true
[AnalysisSettings]
Pairwise Alignment = ====================
DNAPWGapOpeningPenalty = 15.00
DNAPWGapExtensionPenalty = 6.66
Multiple Alignment = ====================
DNAMAGapOpeningPenalty = 15.00
DNAMAGapExtensionPenalty = 6.66
Global Options = ====================
DNA Weight Matrix = IUB
TransitionWeightNEdit = 0.50
UseNegativeMatrix = ON
DelayDivergentCutoff = 30
KeepPredefinedGaps = True

Table S3: infer_ME_nucleotide.mao

[MEGAinfo]	
ver	= 10200331-x86_64 MS Windows
[DataSettings]	
datatype	= snNucleotide
containsCodingNuc	= False
MissingBaseSymbol	= ?
IdenticalBaseSymbol	= .
GapSymbol	= -
Labelled Sites	= All Sites
Labels to Include	=
[ProcessTypes]	
ppInfer	= true
ppME	= true
[AnalysisSettings]	
Analysis	= Phylogeny Reconstruction
Scope	= All Selected Taxa
Statistical Method	= Minimum Evolution method
Phylogeny Test	= ====================
Test of Phylogeny	= None
No. of Bootstrap Replications	= Not Applicable
Substitution Model	= ====================
Substitutions Type	= Nucleotide
Model/Method	= Maximum Composite Likelihood
Substitutions to Include	= d: Transitions + Transversions
Rates and Patterns	= ====================
Rates among Sites	= Uniform Rates
Gamma Parameter	= Not Applicable
Pattern among Lineages	= Same (Homogeneous)
Data Subset to Use	= ====================
Gaps/Missing Data Treatment	= Pairwise deletion
Site Coverage Cutoff (%)	= Not Applicable
Tree Inference Options	= ====================
ME Heuristic Method	= Close-Neighbor-Interchange (CNI)
Initial Tree for ME	= Obtain initial tree by Neighbor-Joining
ME Search Level	= 1
System Resource Usage	= ====================

Number of Threads	= 1
Has Time Limit	= False
Maximum Execution Time	= -1

Table S4: infer_ML_nucleotide.mao

[MEGAinfo]	
ver	= 10200331-x86_64 MS Windows
[DataSettings]	
datatype	= snNucleotide
containsCodingNuc	= False
MissingBaseSymbol	= ?
IdenticalBaseSymbol	= .
GapSymbol	= -
Labelled Sites	= All Sites
Labels to Include	=
[ProcessTypes]	
ppInfer	= true
ppML	= true
[AnalysisSettings]	
Analysis	= Phylogeny Reconstruction
Statistical Method	= Maximum Likelihood
Phylogeny Test	= ====================
Test of Phylogeny	= Bootstrap method
No. of Bootstrap Replications	= 100
Substitution Model	= ====================
Substitutions Type	= Nucleotide
Model/Method	= Tamura-Nei model
Rates and Patterns	= ====================
Rates among Sites	= Gamma Distributed With Invariant Sites (G+I)
No of Discrete Gamma Categories	= 5
Data Subset to Use	= ====================
Gaps/Missing Data Treatment	= Use all sites
Site Coverage Cutoff (%)	= Not Applicable
Tree Inference Options	= ====================
ML Heuristic Method	= Nearest-Neighbor-Interchange (NNI)
Initial Tree for ML	= Make initial tree automatically (Default - NJ/BioNJ)
Branch Swap Filter	= None
System Resource Usage	= ====================
Number of Threads	= 8
Has Time Limit	= False
Maximum Execution Time	= -1

Table S5: infer_MP_nucleotide.mao

MEGAinfo]	
ver	= 10200331-x86_64 MS Windows
[DataSettings]	
datatype	= snNucleotide
containsCodingNuc	= False
MissingBaseSymbol	= ?
IdenticalBaseSymbol	= .
GapSymbol	= -
Labelled Sites	= All Sites
Labels to Include	=
[ProcessTypes]	
ppInfer	= true
ppMP	= true
[AnalysisSettings]	
Analysis	= Phylogeny Reconstruction
Statistical Method	= Maximum Parsimony
Phylogeny Test	= ====================
Test of Phylogeny	= Bootstrap method
No. of Bootstrap Replications	= 100
Substitution Model	= ====================
Substitutions Type	= Nucleotide
Data Subset to Use	= ====================
Gaps/Missing Data Treatment	= Use all sites
Site Coverage Cutoff (%)	= Not Applicable
Tree Inference Options	= ====================
MP Search Method	= Subtree-Pruning-Regrafting (SPR)
No. of Initial Trees (random addition)	= 10
MP Search level	= 1
Max No. of Trees to Retain	= 100
System Resource Usage	= ====================
Number of Threads	= 8
Has Time Limit	= False
Maximum Execution Time	= -1

Table S6: infer_NJ_nucleotide.mao

[MEGAinfo]	
ver	= 10200331-x86_64 MS Windows
[DataSettings]	
datatype	= snNucleotide
containsCodingNuc	= False
MissingBaseSymbol	= ?
IdenticalBaseSymbol	= .
GapSymbol	= -
Labelled Sites	= All Sites
Labels to Include	=
[ProcessTypes]	
ppInfer	= true
ppNJ	= true
[AnalysisSettings]	
Analysis	= Phylogeny Reconstruction
Scope	= All Selected Taxa
Statistical Method	= Neighbor-joining
Phylogeny Test	= ====================
Test of Phylogeny	= None
No. of Bootstrap Replications	= Not Applicable
Substitution Model	= ====================
Substitutions Type	= Nucleotide
Model/Method	= Maximum Composite Likelihood
Substitutions to Include	= d: Transitions + Transversions
Rates and Patterns	= ====================
Rates among Sites	= Uniform Rates
Gamma Parameter	= Not Applicable
Pattern among Lineages	= Same (Homogeneous)
Data Subset to Use	= ====================
Gaps/Missing Data Treatment	= Pairwise deletion
Site Coverage Cutoff (%)	= Not Applicable
System Resource Usage	= ====================
Number of Threads	= 1
Has Time Limit	= False
Maximum Execution Time	= -1
